# Reducing cell wall feruloylation by expression of a fungal ferulic acid esterase in *Festuca arundinacea* modifies plant growth, leaf morphology and the turnover of cell wall arabinoxylans

**DOI:** 10.1371/journal.pone.0185312

**Published:** 2017-09-21

**Authors:** Marcia M. de O Buanafina, Prashanti R. Iyer, M. Fernanda Buanafina, Erica A. Shearer

**Affiliations:** Department of Biology, The Pennsylvania State University, University Park, PA, United States of America; Universidade de Lisboa Instituto Superior de Agronomia, PORTUGAL

## Abstract

A feature of cell wall arabinoxylan in grasses is the presence of ferulic acid which upon oxidative coupling by the action of peroxidases forms diferuloyl bridges between formerly separated arabinoxylans. Ferulate cross-linking is suspected of playing various roles in different plant processes. Here we investigate the role of cell wall feruloyaltion in two major processes, that of leaf growth and the turnover of cell wall arabinoxylans on leaf senescence in tall fescue using plants in which the level of cell wall ferulates has been reduced by targeted expression of the *Aspergillus niger* ferulic acid esterase A (FAEA) to the apoplast or Golgi. Analysis of FAE expressing plants showed that all the lines had shorter and narrower leaves compared to control, which may be a consequence of the overall growth rate being lower and occurring earlier in FAE expressing leaves than in controls. Furthermore, the final length of epidermal cells was shorter than controls, indicating that their expansion was curtailed earlier than in control leaves. This may be due to the observations that the deposition of both ether and ester linked monomeric hydroxycinnamic acids and ferulate dimerization stopped earlier in FAE expressing leaves but at a lower level than controls, and hydroxycinnamic acid deposition started to slow down when peroxidase levels increased. It would appear therefore that one of the possible mechanisms for controlling overall leaf morphology such as leaf length and width in grasses, where leaf morphology is highly variable between species, may be the timing of hydroxycinnamic acid deposition in the expanding cell walls as they emerge from cell division into the elongation zone, controlled partially by the onset of peroxidase activity in this region.

## Introduction

Plants rely on their cell walls to provide shape and strength to cells, to glue cells together and to give rigidity to the whole plant; all of which are of fundamental importance to plant growth and development [1–3]. Plant cell walls are complex structures composed of cellulose microfibrils, noncellulosic polysaccharides, proteins and phenolic substances [4, 2, 5]. The cell walls of grasses are distinctive in composition, with heteroxylans usurping the roles of some of the other matrix polysaccharides in dicots [4]. The xylans of primary walls typically have arabinose and glucuronic acid side chains, and are present as arabinoxylans (AX) or glucuronoarabinoxylans (GAX). A distinct feature of AX in grasses is also the presence of hydroxycinnamic acids, mainly ferulic and *p*-coumaric acids. Ferulic acid (FA) is introduced into the cell wall via an ester linkage at the arabinose O-5 position of the arabinoxylan side chain, which upon oxidative coupling by the action of peroxidases forms diferuloyl bridges between formerly separated AX molecules [6–8]. This creates intra- and intermolecular cross-links between AX backbones. Ferulic acid can also be ether-linked to lignin monomers and oligomers resulting in the linkage of lignin to the xylan/cellulose network via lignin-ferulate-xylan complexes [9–14].

The level of arabinosyl substitution decreases considerably as cells cease elongation and at the same time the percentage of feruloylated arabinosyl units increases [15–16]. Ferulate cross-linking is suggested to play a role in both cell wall assembly [17–18] and in the loss of cell wall extensibility [19] along with a cessation of leaf growth [20–23] by cross-linking cell wall components, which also hinders the rate and extent of cell wall degradation by ruminant microbes [24] and fungal enzymes [25]. Ferulates have also been implicated in plant resistance to pathogens [26–28] by cross-linking cell wall components.

The specific role of feruloylation in these various processes has been established largely by indirect experiments. Kamisaka *et al*. (1990) [20] reported the correlation between the level of cell wall ferulates of *Avena* coleoptiles and mechanical stiffness of their cell walls. Likewise in tall fescue leaves, a deceleration in the leaf elongation was correlated with the formation of cell wall ester linked ferulate dimers [23]. Similar results were also reported in submerged floating rice internodes [29]. Although these studies reached a similar conclusion about the potential importance of wall feruloylation, they depend on correlations between two processes but do not actively manipulate the patterns of wall feruloylation and examine the consequences for cell growth and cell wall properties. Buanafina *et al*. (2010) [30] have previously shown that intracellular targeted expression of a *Aspergillus niger* ferulic acid esterase (FAE) to the apoplast or Golgi in tall fescue was a successful strategy for decreasing the level of cell wall ferulates and diferulates, which targets the inter-chain interactions as the cell wall polymers are being formed.

Feruloyl esterases are a type of carboxylic acid esterase that can hydrolyze the 1->5 ester bond between the ferulate and arabinose in the cell wall [31] thereby releasing the ferulates and diferulates from the cell wall [32]. This should provide a stronger test of the hypothetical roles for wall feruloylation than has been feasible previously. In fact, it has allowed us to test the direct role of ferulates on cell wall degradability [33, 34, 30, 35], and plant resistance [36].

The present study is aimed at investigating the role of cell wall feruloyaltion in two major processes, that of leaf growth and arabinoxylan turnover in tall fescue. This has been accomplished using transgenic plants where the level of cell wall ferulates has been specifically reduced by targeted expression of the *Aspergillus niger* FAEA to the apoplast or Golgi.

In previous work, Golgi and apoplastic targeted expression of *Aspergillus niger* FAE showed little effect on growth and development in the T0 generation [30]. However, repeated vegetative propagation of these plants via tillering was found to result in a new and stable phenotype after 4–5 generations, characterized by narrower and shorter leaves, altered leaf growth kinetics and shorter roots [37]. The plants under study in this work correspond to the T8 through T10 generations of vegetative propagation. Similar changes in leaf morphology following repeated vegetative propagation were also apparent in an additional set of plants expressing FAE and xylanase [37].

By isolating cell walls from leaf blades at different developmental stages and quantifying the level of ferulates, arabinose and xylose, we have been able to associate the reduction in the levels of cell wall ferulates and sugar composition with changes in leaf morphology and in the growth rate of leaves and roots, providing direct evidence for a significant impact of ferulates in leaf-elongation and cell wall sugar turnover, starting from very early stages of plant growth.

## Material and methods

### Plant material and growth conditions

Five lines of tall fescue vegetatively propagated via tillers over eight generations, were included in this study: a non-transgenic control line (C) and four transgenic lines expressing FAEA. Two of the lines had FAEA targeted to the apoplast (T27 and T27R) and two lines had FAEA targeted to the Golgi (T28 and T29). Transgenic plants were produced by particle bombardment of immature embryogenic suspension culture of *Festuca arundinacea* genotype 20BN3 from cultivar S170, with two gene constructs carrying a genomic clone of *Aspergillus niger faeA*. These vectors were made in the pCOR105 plasmid under the rice actin promoter with the rat sialyl transferase motif added to confer Golgi targeted FAEA expression or with the potato protease inhibitor II (PPI) apoplast motif added to confer apoplast targeted FAEA expression as previously described [34, 30]. The control (non-transformed) genotype used in all the experiments was also derived from tissue culture of genotype 20BN3. Characterization of these transgenic plants at the whole plant level including gene integration and the effect of targeted FAEA expression on cell wall ester-linked ferulates, sugars, and lignin composition has been reported previously [30].

Clonal tillers of each of the five *Festuca* lines were separated from established mother plants, planted in 2” pots, grown for 2 weeks and then transferred to 6” pots containing a 5:1 mixture of Miracle-Grow potting mix (The Scotts Company, Marysville, OH 43041) and vermiculite, and grown in a controlled environmental chamber at 22°C/16°C (day/night) temperature, under a 16 h photoperiod and light intensity of 180 μmol.m‾^2^.s‾^1^. Three pots of each of the five lines were randomly arranged in one growth chamber for the duration of the experiments, watered daily and fertilized at four-week intervals.

### Growth measurements

#### Leaf growth rates

Leaf growth rates were calculated from the third emerging leaf from replicate tillers (T8-T9 generations) as the mean daily increase in leaf length, until leaves stopped elongating at 30–40 days.

*Segmental Elongation Rate*s were determined by directly piercing the whorl of leaf sheaths with a fine needle above the thickening of the tiller (T9- T10 generations) region associated with the vegetative tiller terminal meristem, so that the lowest pin prick was placed close to the origin of the elongating leaf blade, close to the ligule. Thirteen pin pricks were made 3 mm apart placed parallel to the tiller. This 42-mm interval included the elongation zone. After six hours, the tillers were removed from the plant, dissected to expose the elongating leaf and the distances between pin pricks were measured. Relative Segmental Elongation (RSE) rates were calculated from displacement of the pin pricks, where RSEi = 2(ditn–di,t0)* (di,tn + di,t0) -1 where di,t0 is the distance between the neighbouring pin marks for segment I at the time of making the mark (t0) and di,tn is the distance between the marks after a period of (tn-t0) following growth. RSEi values were converted to percentage values, for data comparison as follows: % RSEi = 100 RSEi * (RSE1 + RSE2 + …RSEn)-1 where n is the total number of segments in the elongation zone.

### Epidermal cell number and dimensions

The number of epidermal cells per unit area and the spatial distribution of cell lengths and widths were determined from replicas of abaxial epidermal peels of fully extended leaf blades (T9- T10 generations), taken 1cm from the leaf tip in rows adjacent to the leaf midrib, from 3 replicates of each of the five lines. Samples were prepared as described in Weyers and Travis *et al*. (1981) [38]. Peels were stained briefly in 0.05% (w/v) solution of toluidine blue in 0.1 M potassium phosphate buffer [39], destained in double distilled water and placed on a glass slide cuticle side up, with a drop (5–10μL) of 50% glycerol. Area (lengths and widths) of cells in rows adjacent to the midrib, were measured on freshly prepared peels under an Olympus IX51 light microscope attached to an Olympus XC10 camera under 20 x lenses and analysed with cellSens Entry Software. Ten measurements were recorded for each peel, corresponding to one replication, and a total of 10 different leaves were measured and recorded per line. The number of cells was also counted within 150000 μm^2^ areas, from three different leaf zones (base, middle and tip) from five leaves of the control and the T27R line.

### Tissue sampling

Elongating tiller leaves (from T8- T9 generations) were selected for sampling when the youngest blade was 14–16 cm long. The inner leaf was carefully freed from the surrounding leaf sheaths, and cut from the base at the ligule to the tip. Starting from the beginning of the elongation zone, the leaf blades were cut into ten sequential 10-mm-long segments. To measure FAE activity along leaves, 25–30 segments were pooled by location along the leaf blades, which represented one replication, with two to three replications per plant line. To measure HCA levels along the leaf, 120–130 segments were pooled by location along the leaf blade as one replicate with 2–3 replications per line. For peroxidase activity assays, leaf blades were cut into sequential 5-mm-long segments and 8–10 segments were combined per replicate. Segments were collected in tared microfuge tubes and fresh weights determined. Segments were frozen in liquid nitrogen, ground to a fine powder and stored in -80°C for latter assays. To measure HCA levels in roots, whole primary roots from hydroponic grown tillers were harvested before the formation of secondary roots.

### Determination of peroxidase activity

Soluble and ionically bound peroxidase (POX) activities were determined in soluble protein extracts from elongating leaf segments (from T9- T10 generations) as in Sandoya and Buanafina (2014) [40] with minor modifications. Briefly, leaf segments were ground to a fine powder in liquid nitrogen and soluble peroxidase extracted with 150 μl per 50mg fresh tissue of 0.5% PVP in 0.1 M potassium phosphate buffer (pH 7). Suspension was left in ice for 5 min and centrifuged at 11,000 × g for 10 min at 4°C. To extract ionically-bound peroxidase, the pellets from above were mixed with the same buffer containing 0.8M KCl [41]. The clear supernatants from each extraction were collected and POX enzyme activities were measured using a substrate mixture containing 926 μl of 3 mM guaiacol and 25 μl of 1 mM H_2_O_2_ added to 25 μl of enzyme extract. Changes in absorbance at 450 nm were recorded in a Thermo/Spectronic 21D Milton Roy^®^ spectrophotometer after 5 min and POX activity was expressed as the change in absorbance at 450 nm (Δ*A*_450_) per mg fresh weight.

### Determination of FAE activity

Freshly harvested leaf blade segments from each replication were frozen in liquid nitrogen, ground to a fine powder and soluble proteins extracted with 0.1 M Na acetate, pH 5.5 buffer. Extracts were incubated at 28°C for 24hrs, with 24 mM ethyl ferulate as substrate and FAEA activities determined from the released ferulic acid by high-performance liquid chromatography (HPLC) as in Buanafina *et al*. (2012) [35]. One unit of FAEA activity equals 1 μg ferulic acid released from ethyl ferulate in 24 h at 28°C.

### Cell wall analysis

#### Preparation of isolated cell walls (AIR)

Leaf and root tissues were harvested as described in the previous section, freeze-dried and milled to a fine powder and cell walls were prepared as described in Buanafina *et al*. (2015) [37].

#### Determination of cell wall ether and ester-linked hydroxycinnamic acids

Quantitative analysis of ester-linked HCAs were performed on determined isolated cell walls (AIR) following treatment with 1M NaOH at 25°C for 20h, as described in Buanafina *et al*. (2015) [37] but with 2-hydroxycinnamic acid (0.1 mg) added as an internal standard.

Measurements of total ether+ester linked HCAs were determined as in Grabber *et al*. (1995) [42] with some modifications as follows: AIR samples (20–30 mg) were weighted into 10 ml Teflon vessels (PTFE) and incubated with 3 ml of 4M NaOH and vessels were purged with N_2._. 3-hydroxy-4-methoxycinnamic acid (0.1 mg) was added as an internal standard. Vessels with samples were placed inside Teflon bomb (Parr Instrument Co.) containing 3–4 ml water. The bomb was placed into a forced air oven at 170°C for 3 hours. Samples were allowed to cool and total HCAs separated as previously described [35].

#### Determination of the cell wall sugars arabinose and xylose

The levels of the monosaccharides arabinose and xylose in isolated cell wall AIR were determined by High Performance Anion Exchange Chromatography (HPAEC) of hydrolysed cell wall samples based on the method of Øbro *et al*. (2004) [43] with modifications as described in Buanafina *et al*. (2012) [35].

### Anatomical analyses

Transverse sections (2mm x 5mm) from the youngest leaves (T9- T10 generations), 10 cm distal to the ligule were taken and prepared according to Owen and Makaroff (1995) [44] with minor modifications. Samples were placed in a solution of 2.8% (v/v) glutaraldehyde in 0.1 M HEPES buffer, pH 7.2 and 0.02% (v/v) Triton X-100 and infiltrated with 1h vacuum cycles for 8 hours and then fixed overnight in the same buffer at 4°C. Samples were rinsed three times for 15min in the same buffer and post-fixed in 1% (w/v) aqueous OsO_4_ overnight at 4°C in the dark. Samples were dehydrated in a graded acetone series and infiltrated with resin (Spurr’s): acetone. The samples were then embedded in pure Spurr’s resin and allowed to polymerize at 60°C for 48 hours. Samples were trimmed with a razor blade and 0.5 μm transverse sections cut with a Leica Ultra microtome equipped with a glass knife and a clearance angle of 4°. Sections were stained for 20–30 seconds in a 0.1% (w/v) aqueous solution toluidine blue and placed on a hot block (50°C) to dry. Samples were viewed on an Olympus IX51 light microscope and photographed using an Olympus XC10 encased in a U-CMAD3 digital camera.

### Hydroponic plant growth

Clonal tillers from control and transgenic plants (T8- T9 generations) were separated from mother plants and placed in holes drilled in the lids of 11 L plastic containers used as hydroponic tanks which contained rockwool as support. The tanks were filled with 7.5L of water containing dilute liquid grow nutrition solution (Dyna-Grow, Richmond, CA, 1:5000 dilution). The hydroponics tanks were maintained in a controlled environmental chamber at 22/16°C (day/night) temperature, 16 h light period. The roots were constantly aerated using an aquarium air pump (AQUA culture). Root lengths were measured prior to emergence of secondary roots in the control plants, and both control and transgenic roots were harvested for determination of HCA content in the primary roots before formation of secondary roots.

### Chlorophyll determinations

Leaf growth of transgenic and control plants (T8-T9 generations) growing in 6” pots was measured daily and leaves were marked for harvest every 5 days. For each time point: leaf blades were excised, weighed to obtain 0.2 g fresh weight and sliced into 1mm wide sections. Duplicate samples were stored at -80°C until analysis. Chlorophyll was extracted from fresh (or frozen) material harvested from 1 to 100 days following leaf emergence, with three leaves being harvested at each stage, from the control and the FAE expressing T27R lines.

All extractions were performed at 4°C under low light, in 10 ml of ice-cold 80% aqueous acetone, centrifuged at 3000 rpm for 10 minutes and chlorophyll absorbance measured between 620 and 700 nm on a spectrophotometer against an 80% acetone blank. Absorbance at 645, 652 and 663 nm was determined from the spectrum and chlorophyll concentration calculated according to Morris (1997) [45].

### Statistical analysis

Statistical Analysis System (SAS) [46] (2015) software version JPM Pro 12.1.0 (SAS Institute Inc., Cary, NC) was used for all statistical analysis. Values in the text are means ± standard error of the mean (SEM). Bars with different letters are significantly different (Tukey’s, α = 0.05).

## Results

### FAE activities in transgenic plants

#### FAE activity in leaves of different ages and in mature roots

**Fig 1:**
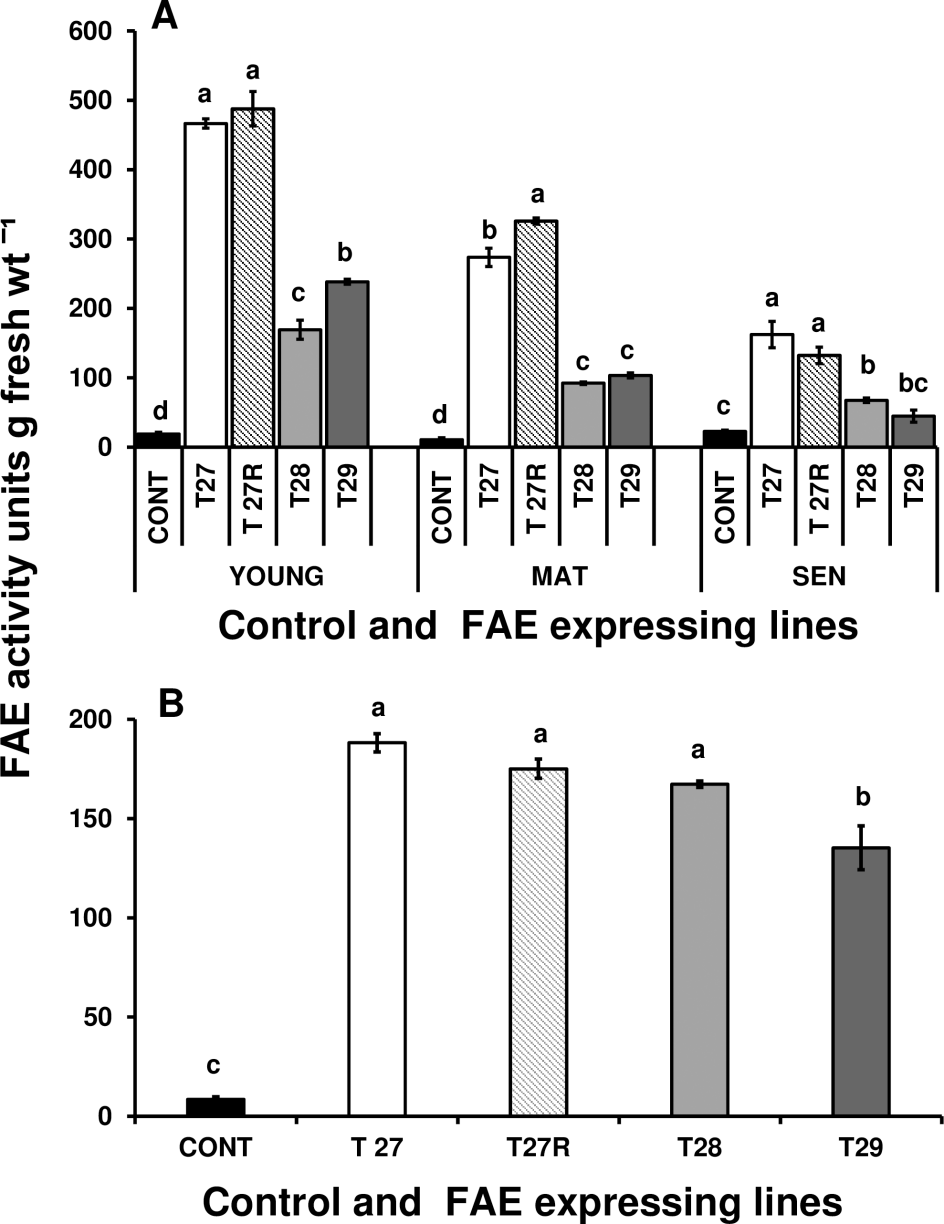
Levels of FAEA enzyme activity in whole young, mature and senescing leaves (A) and in whole mature roots (B) of control, apoplast (TR27, TR27R) and Golgi (T28 and T29) FAE expressing plants. Mean ±SEM (n = 3–5). Different letters indicate significant differences from the control (Tukey’s = 0.05). One unit of FAEA activity equals 1 μg ferulic acid released from ethyl ferulate in 24 h at 28°C.

In agreement with previous results [34, 30], the FAE activity in plants from tillering rounds T8 to T9 was also higher under apoplast targeting than under Golgi targeting. FAE activity was also found to vary with leaf age in all the FAE expressing lines under study, with the highest activity found in young leaves, which decreased by 50% as leaves matured, and by a further 50% as leaves senesced (Fig 1A). The FAE activities in roots of mature plants were less dependent on the targeting sequence but were significantly higher than controls (P < 0.0001) (Fig 1B).

#### FAE activity along the leaf blade including the expansion zone

**Fig 2:**
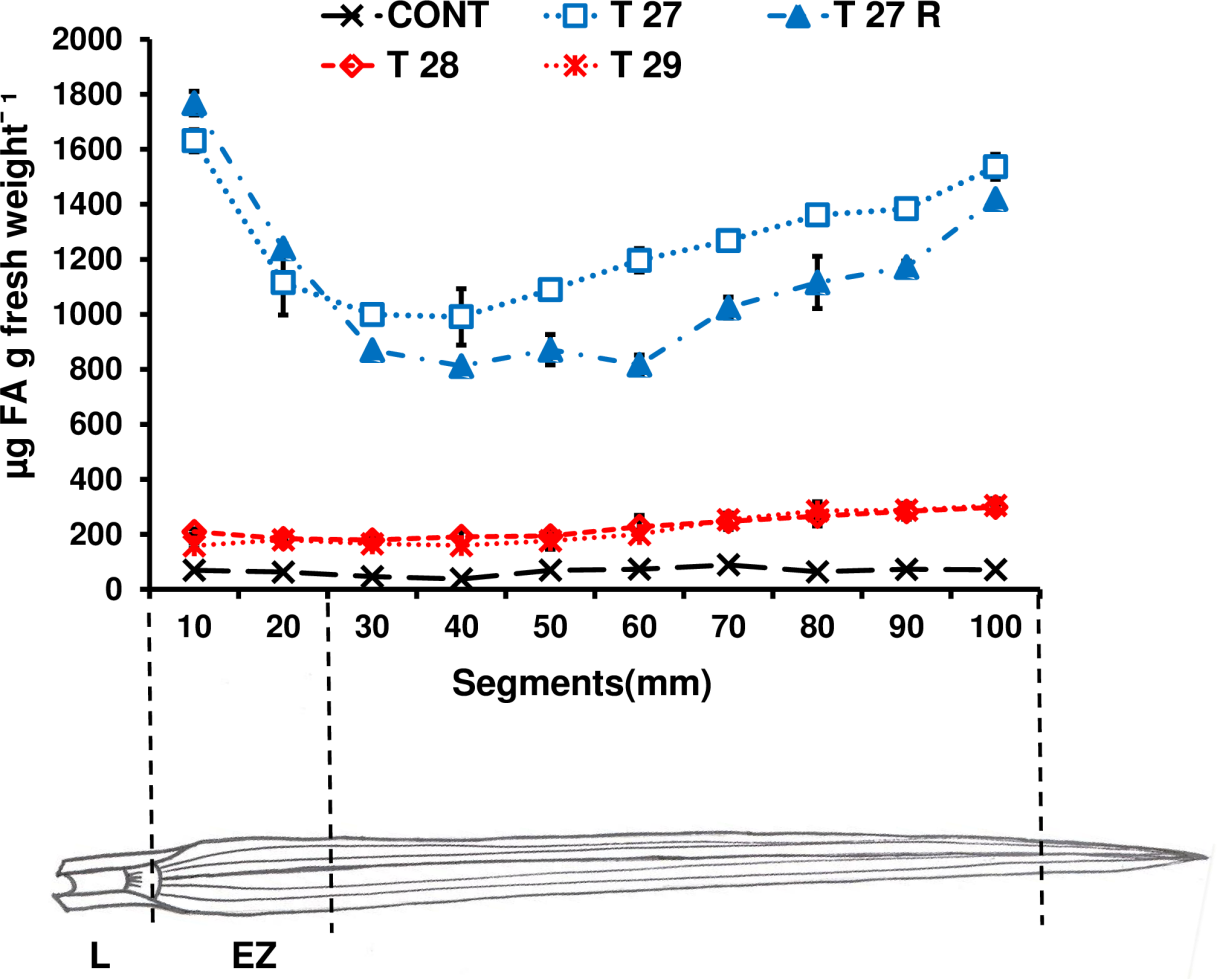
Levels of FAE enzyme activity in 10 mm segments along 12–14 cm leaf blades starting at the cell elongation zone of control, apoplast (T27 and T27R) and Golgi (T28 and T29) FAE expressing plants. Mean ± SEM (n = 2–3 replicates of 25–30 leaf sections from two independent transformed plants). L = ligula; EZ = extension zone. One unit of FAEA activity equals 1 μg ferulic acid released from ethyl ferulate in 24 h at 28°C.

To confirm that plants of the T9-T10 generation were still actively expressing FAE, the activity of extracts of the youngest expanding leaves within tillers were measured. FAE activities in both lines where FAE was targeted to the apoplast varied along the leaf blades and were highest in the first 10mm, corresponding to the cell expansion zone, declined in the next 30–40 mm, and then increased linearly up to 100 mm at the leaf tip (Fig 2). In contrast, the FAE activity in the leaves of both lines where FAE was targeted to the Golgi was much lower than in leaves where FAE was targeted to the apoplast, and maintained an almost constant level from the first 10mm to 50–60 mm, and then gradually increased to the leaf tip (Fig 2). As expected, only nonspecific background activity was found in tissue-cultured, non-transformed control leaf extracts (Fig 2).

Higher FAE activities in plants with the N-terminal apoplast targeting motif from the protease inhibitor (PPI) (T27, T27) and lower activities in plants with the Golgi targeting motif (T28, T29) were in agreement with previous results [30].

### Morphological phenotypes of FAE expressing plants

#### Leaves

**Fig 3:**
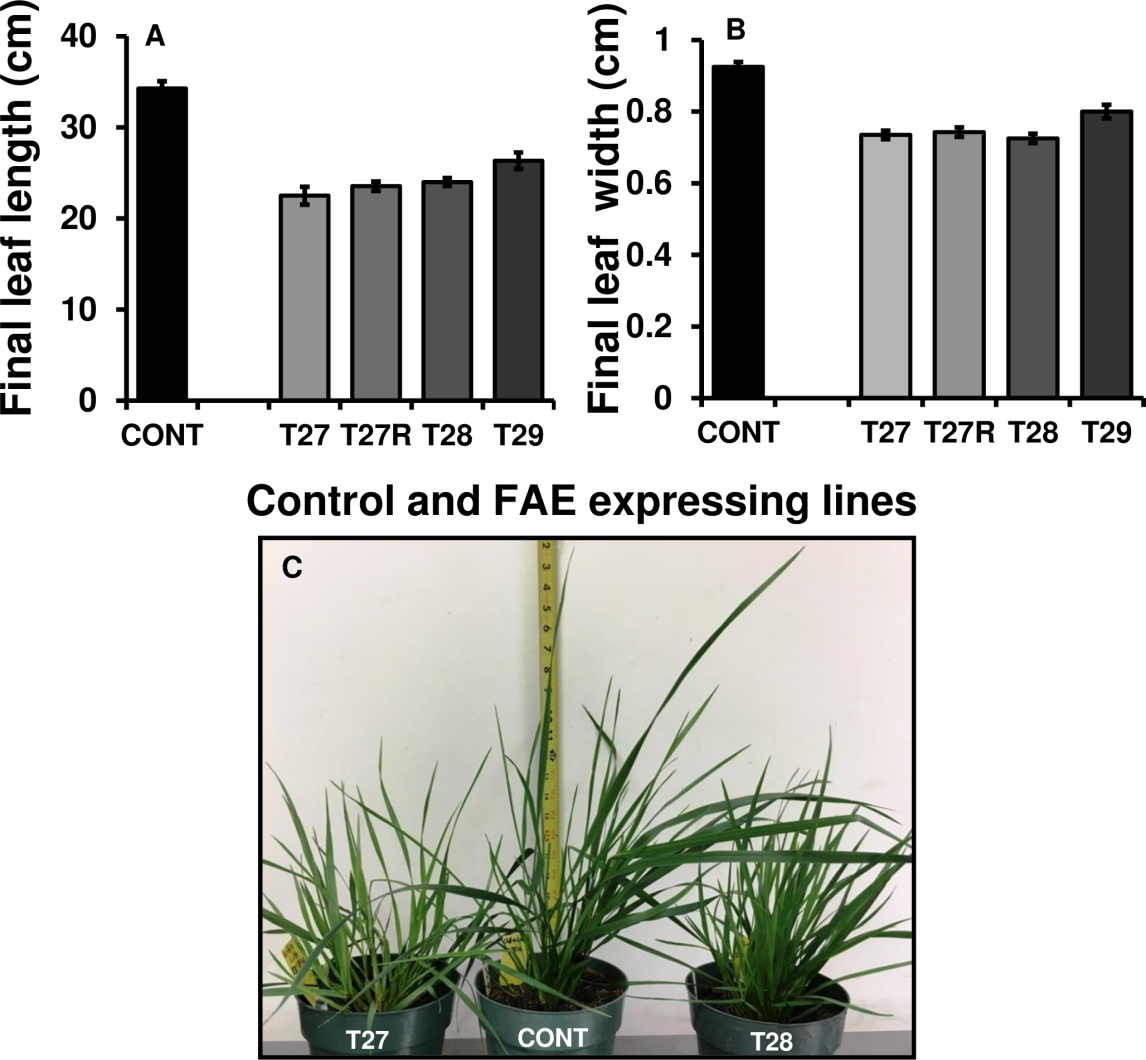
Final leaf length (A) and leaf width (B) of control, apoplast (T27, T27R), and Golgi (T28 and T29) FAE expressing plants. Mean ± SEM (n = 10–15 leaves from individual plants), and plant phenotype (C) of twenty week old control (CONT) and apoplast (T27) and Golgi (T28) FAE expressing plants.

FAE expressing plants were found to have leaves that were 25–35% shorter and 14–22% narrower than control leaves (Fig 3). The maximum length of control leaves was 39 cm (average 34.28 ± 0.91) attained at about the 24^th^ day from leaf emergence, whereas the maximum length of FAE expressing leaves varied from 24.0 cm in plant T27 to 26.0 cm in plant T29 (Fig 3A) attained between the 12^th^ and 20^th^ day from leaf emergence. The overall phenotype of apoplast and Golgi FAE expressing plants compared with the control is shown in Fig 3C.

An important additional observation was that while control leaves remained in the young-rolled stage until they reached three-quarters of their total length, FAE expressing leaves unrolled after 3–4 days of growth, while they were still very young, reaching an advanced developmental stage while the control leaves were still unrolled, which may explain the longer length of the latter and the earlier onset of senescence in the former.

#### Roots

**Fig 4:**
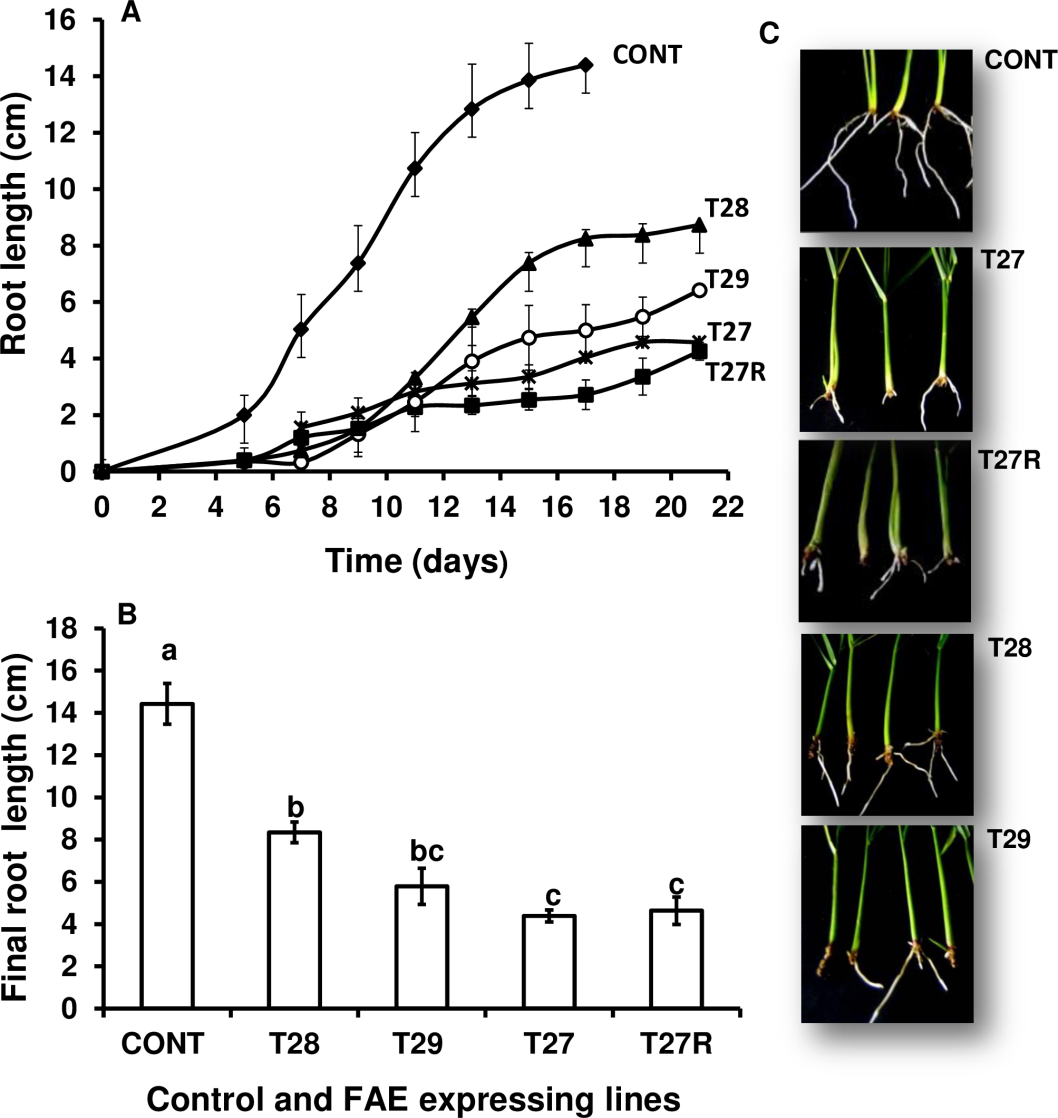
Root growth kinetics (A), final root length (B) and root morphology phenotypes (C) of tillers of control, apoplast (T27 and T27R) and Golgi (T28 and T29) expressing plants grown in hydroponic culture for three weeks. Mean ± SEM (n = 3–5). Letters indicate significant difference (Tukey’s, α = 0.05) among mean values.

The differences observed in the leaf morphology and growth rates between control and FAE lines were also reflected in root characteristics (Fig 4). Root growth rates and root lengths were reduced in hydroponically grown FAE expressing tillers by 70% in apoplast plants T27 and T27R and by 50% in Golgi plants T28 and T29. After 13 days in hydroponic culture, roots from FAE lines did not grow beyond 4–5 cm long, whereas control roots were 14.0 cm long (Fig 4). In addition, by day 20 control roots developed secondary roots and this was not observed in FAE expressing lines.

#### Epidermal cell number and dimensions

**Fig 5:**
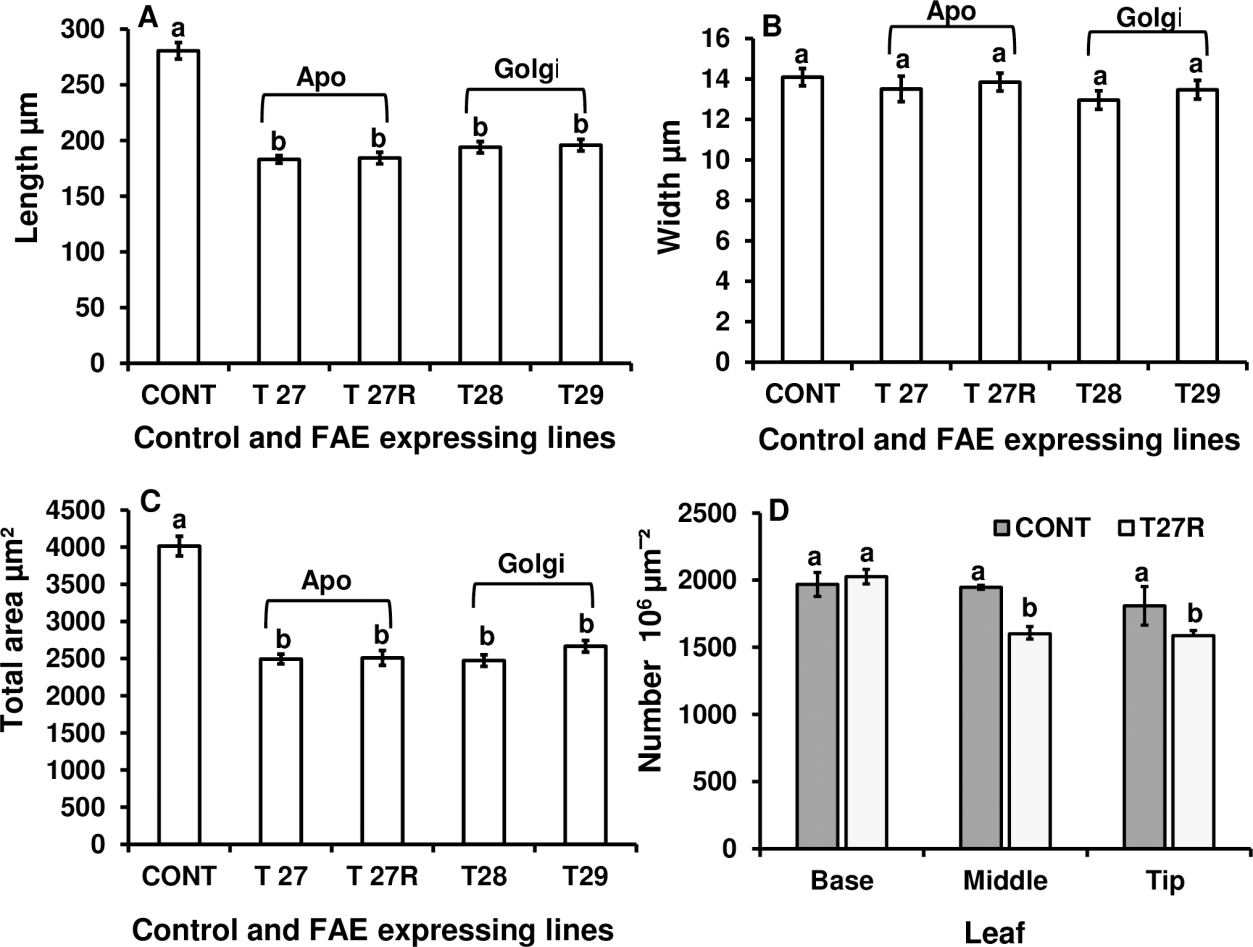
Average epidermal cell length (A), width (B), area (C) and cell number per unit area at the base, middle and tip (D), of the abaxial epidermis of fully expanded leaves of control and apoplast (T27, T27R) or Golgi (T28 and T29) FAE expressing plants. Mean ± SEM n = 10 replicates of 10 leaves per plant, 1 cm from leaf tip in rows adjacent to the leaf midrib. Letters indicate significant difference (Tukey’s, α = 0.05) among mean values.

The reduced leaf length of FAE expressing lines, lead us to examine their anatomical features. Measurements of the length and width of epidermal cells revealed that cells in FAE expressing lines were significantly shorter, by 30% in Golgi and 35% in apoplast compared with controls (P < 0.0001). Cell length varied from 183 to 195 μm compared with 280 μm in control leaves, but were of similar width (P = 0.51) (Fig 5A and 5B) (see also S2 and S3 Tables), resulting in an overall reduction in epidermal cell area of 25% (P < 0.0001) in all four lines (Fig 5C) and could reflect the shorter leaves in the transgenic lines. Preliminary epidermal cell number data between leaves of FAE line T27R and control leaves also showed a small reduction in cell numbers per unit area of leaf in T27R by 18% at 50% leaf length and by 13% at the leaf tip (Fig 5D; S4 Table).

### Growth and developmental phenotypes of FAE expressing plants

**Fig 6:**
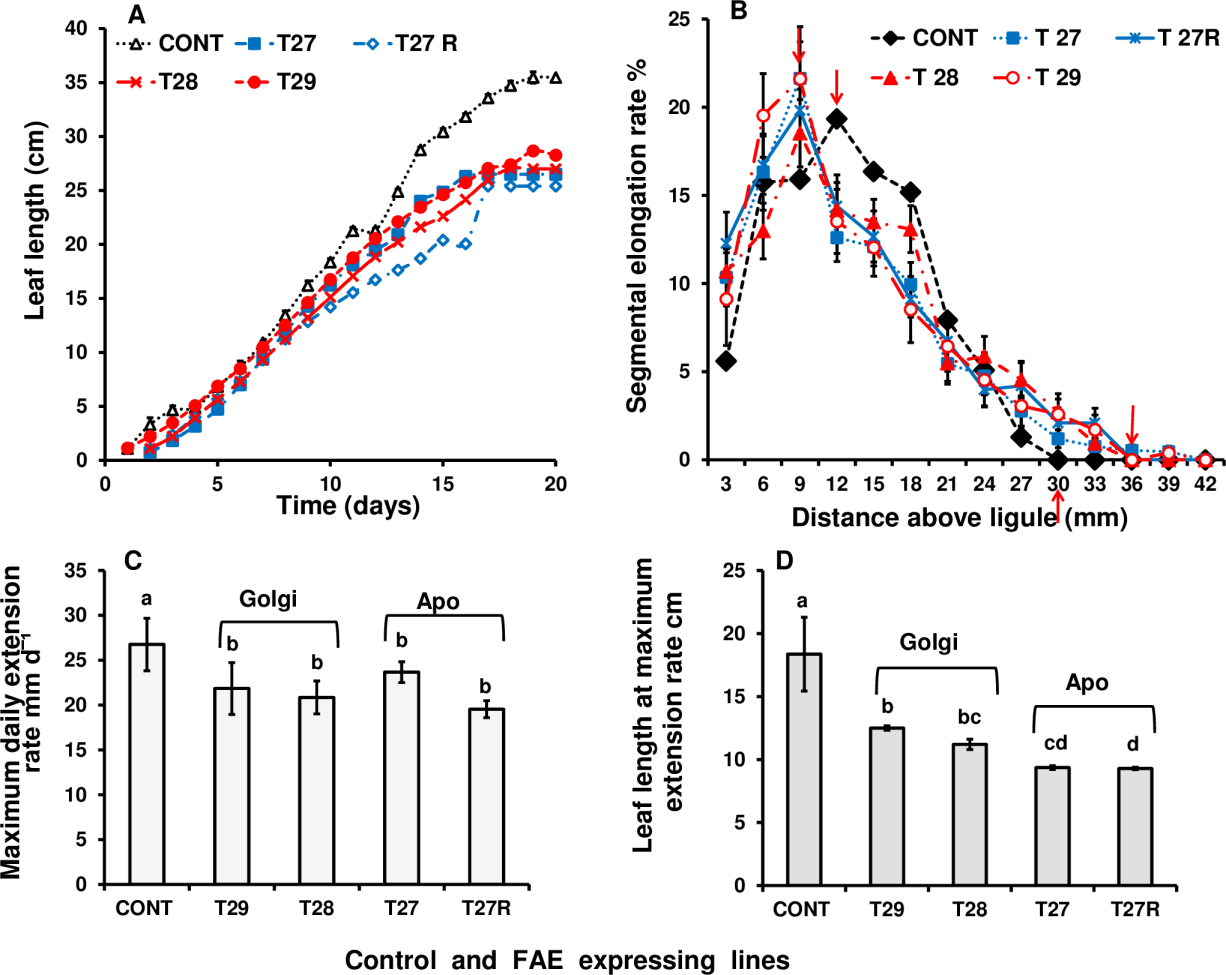
Comparison of growth of newly emerging third leaves of control and apoplast (T27 and T27R) or Golgi (T28 and T29) FAE expressing plants. Growth as increase in leaf length (A), distribution of growth within the elongation zone of leaf blades, determined as the relative segmental elongation rate (B), maximum daily extension rate (C), and leaf length at maximum extension rate (D). Mean ± SEM (n = 20–30 from each plant). Third leaves from tillers of 2–3 plants per line were measured until leaf length was constant. Letters indicate significant difference (Tukey’s, α = 0.05) among mean values.

The growth of newly emerging third leaves, measured as the increase in leaf length differed significantly between control and both apoplast (T27 and T27R) and Golgi (T28 and T29) FAE expressing plants (Fig 6A). Maximum growth rates were lower in both apoplast and Golgi FAE expressing leaves (~19–25 mm/d in apoplast and ~ 21–22 mm/day in Golgi) than in the control leaves (~27 mm / day), (Fig 6C) and occurred when leaves were significantly shorter in both apoplast (at 93–119 mm leaf length) and in Golgi (at 112–125 mm leaf length) FAE expressing leaves than in controls (at 184 mm leaf length) (Fig 6D). However, the maximum relative segmental elongation rates (calculated using the method of Schnyder *et al*. (1987) [47] were higher in both apoplast and Golgi FAE expressing leaves (22%) than in control leaves (19%) and occurred closer to the ligule (at 9 mm) than in controls (at 12 mm) (Fig 6B).

#### Leaf senescence

**Fig 7:**
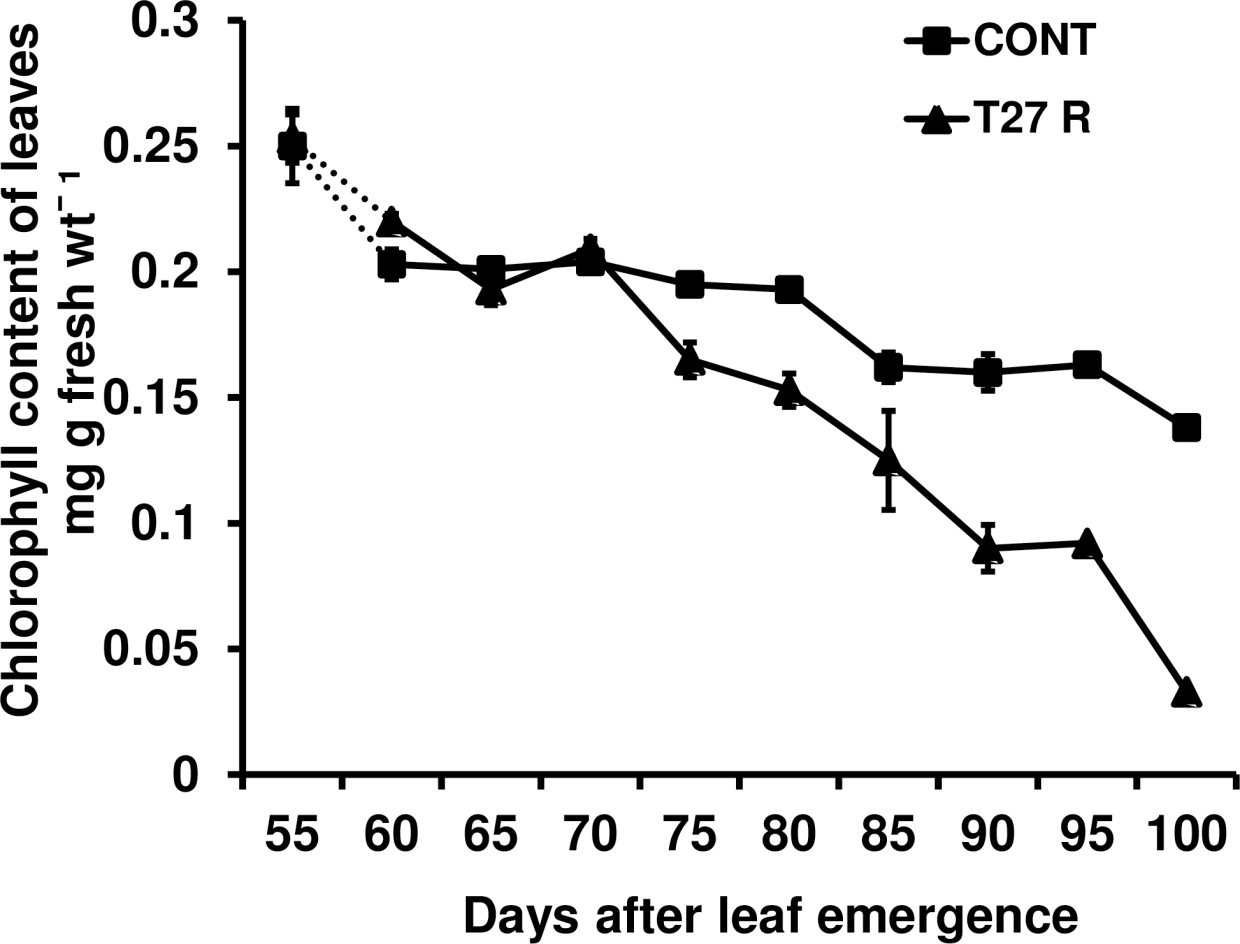
Chlorophyll content of leaves of control and an FAE expressing plant (T27R) during leaf senescence.

To confirm visual observations of an earlier onset of senescence in FAE expressing plants, which reached a more advanced developmental stage, while the control leaves were still growing, the chlorophyll content of leaves of the FAE expressing plant T27R was compared with control leaves. The chlorophyll content of leaves was similar between the control and T27R up to day 70 following leaf emergence but declined faster than in control leaves in the following 30 days, indicating an earlier onset of senescence in FAE expressing plants (Fig 7).

### Chemical phenotypes of FAE expressing plants

#### Variation in the levels of ester and total linked ferulates along leaf blades

**Fig 8:**
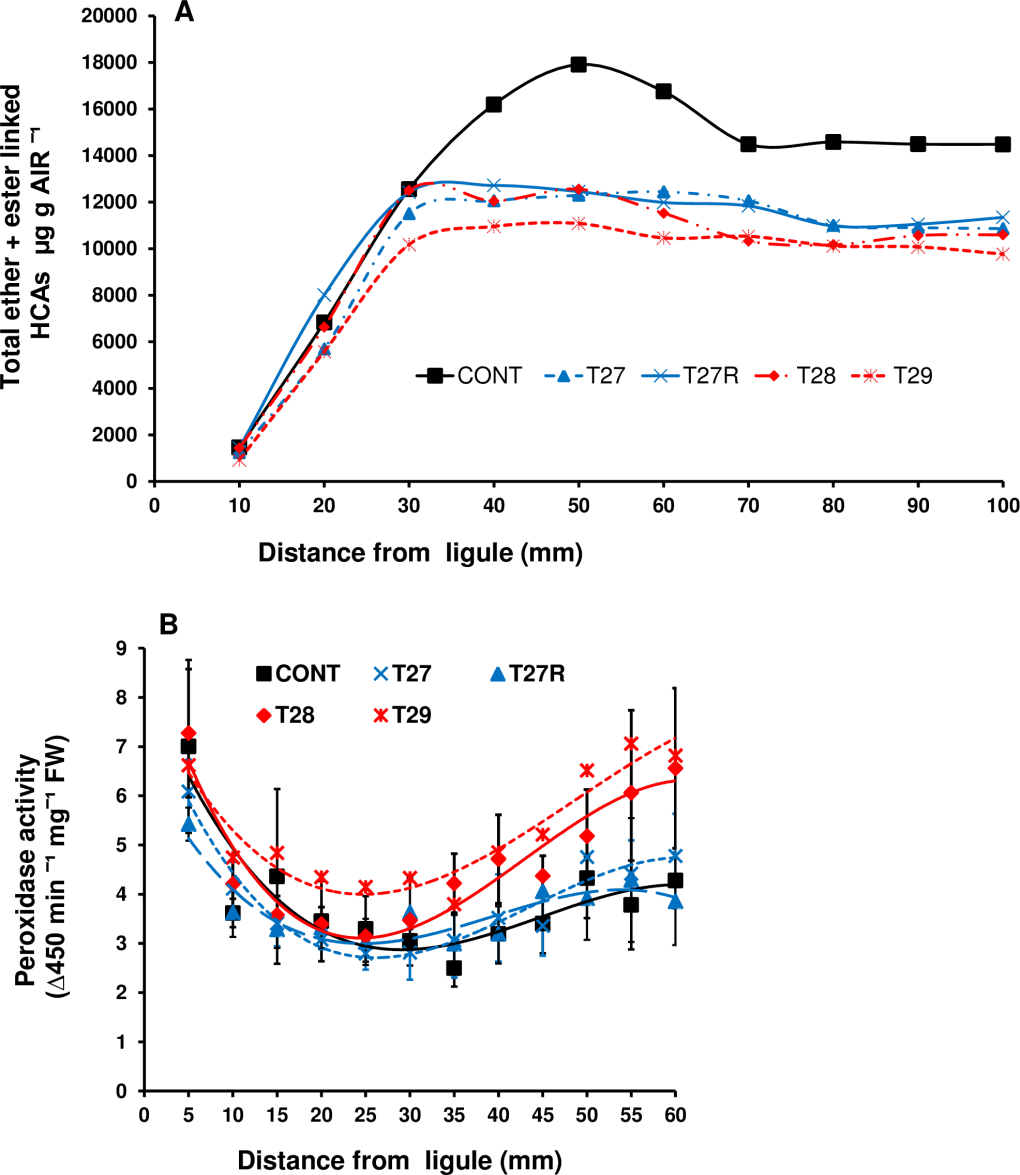
Total ester+ether linked HCAs in cell wall AIR isolated from 1cm leaf sections (A), total (soluble and ionically bound) peroxidase activity (B) in 5mm sections along 14–16 cm leaves starting from the elongation zone of control and apoplast (T27 and T27R) or Golgi (T28 and T29) FAE expressing plants. Leaf sections (120–130) were pooled by location along the leaf blade from 8–9 tillers of 2 to 3 replicates per line from 2 independent FAE expressing plants.

In order to asses if the changes in growth and development were a consequence of FAE expression, the total level of ester and total linked HCAs in cell walls isolated from 10 mm sections along leaf blades including the expanding zone of FAE expressing plants were, compared with control leaves. Total HCAs levels were lower, by 25 to 35%, and plateaued earlier in both apoplast and Golgi FAE expressing lines (Fig 8A).

**Fig 9:**
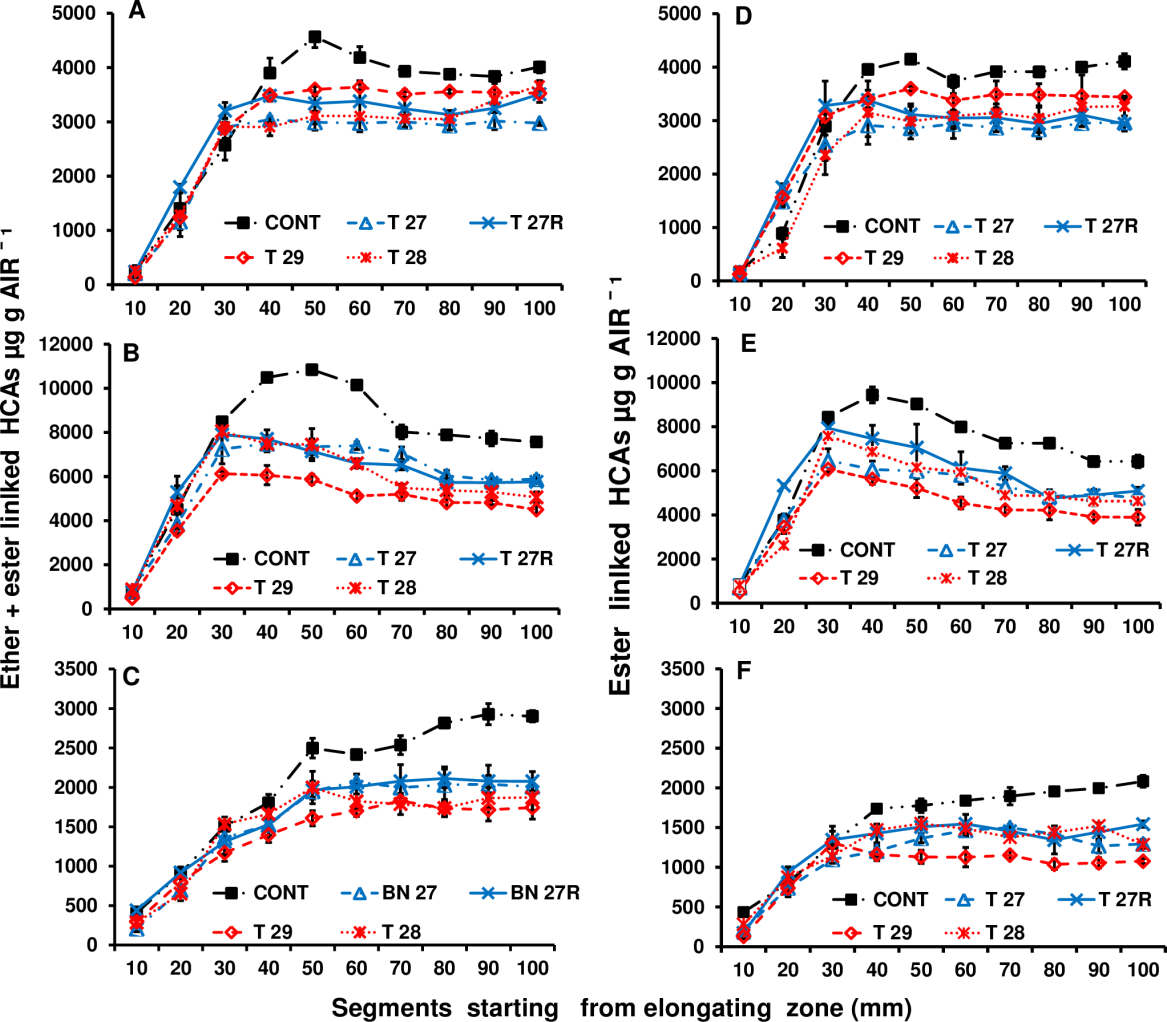
Ester + ether linked HCAs (A-C) and ester linked HCAs (D-F) in cell wall AIR isolated from 10mm segments along 14–16 cm leaves starting from the elongation zone of control and apoplast (T27 and T27R) or Golgi (T28 and T29) FAE expressing plants. *p-*coumaric acid (A, D), ferulate monomers (B, E), and total ferulate dimers (C, F). Leaf sections (120–130) were pooled by location along the leaf blade for each of 2 to 3 replicates per line. Ferulate monomers = trans-ferulic + cis-ferulic acid. Ferulate dimers = 8-0-4’-diferulate + 5–5’diferulate + 8–5 cyclic diferulate (benzo form) + 8–5’-diferulate + an unknown ferulate dimer. Mean ± SEM (n = 4–6 from 2 independent FAE expressing plants).

Total ester+ether linked *p*-coumaric acid levels were max at 50 mm from the ligule and at a higher level (4.5 mg g^-1^ dry weight AIR) in control leaves than in leaves of FAE plants, where this occurred 30 mm from the ligule and at a lower level (3.3 mg g^-1^ dry weight AIR) (Fig 9A). This effect was less pronounced with ester linked *p*CA (Fig 9D).

Deposition of monomeric ferulates behaved in a similar way as *p*CA. Ester+ether linked monomers were at the maximum level at 50 mm from the ligule and at a higher level of 11 mg g^-1^ dry weight in controls than in FAE plants where this occurred at 30 mm from the ligule and at a lower level of 7 to 7.5 mg g^-1^ dry weight (Fig 9B).

Both total linked and ester linked ferulate dimerization had ceased by 50 mm from the ligule, in both apoplast and Golgi FAE expressing leaves but at a lower level compared with control leaves, where dimer formation continued to increase but at a slower rate, to 100 mm from the ligule (Fig 9C and 9F). The level of both ferulate monomers and dimers in segments 10 to 30 mm from the ligule were quite similar between control and FAE expressing lines; however from 30 mm onwards, ferulate levels continued increasing in control leaves but ceased in all the FAE lines.

#### Peroxidase activity in leaves

As FAE expressing lines had narrower leaves compared with control leaves, soluble and cell wall bound peroxidase activities were expressed as a function of fresh weight. Soluble peroxidase activities were highest at the leaf base and decreased with distance for the first 35 mm from the ligule in control leaves and for 25 mm in FAE expressing leaves and then began to increase (S1A Fig) just before the point where the relative segmental elongation rates were zero (see Fig 6B). Ionically bound cell wall peroxidase activities were much lower than the soluble activities and were initially similar in control and FAE expressing leaf bases but increased with distance along the leaf, particularly in the apoplast FAE expressing lines (S1B Fig). It is also worth noting that total HCA deposition began to slow down when peroxidase levels increased (Fig 8A and 8B). The ratio of dimer/monomer ferulic acid was statistically correlated (Sperman’s) with peroxidase activity for control (P = 0.05) but not for FAE expressing lines.

#### Variation in the levels of ester linked ferulates in mature roots

**Fig 10:**
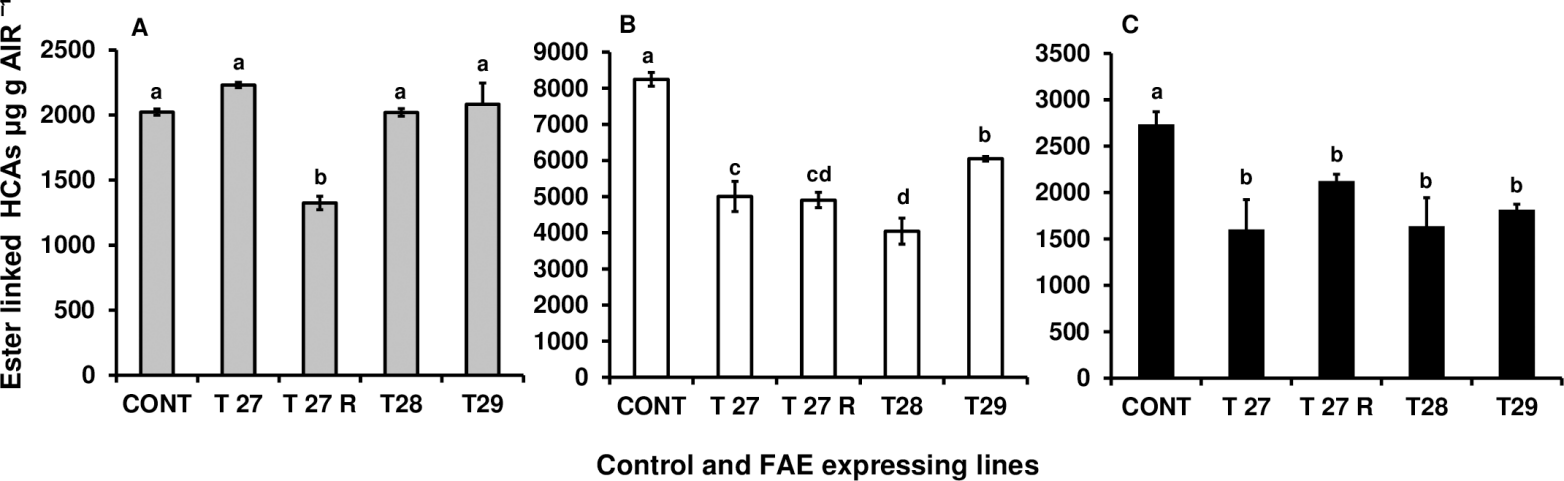
Ester linked HCAs in mature roots. *p*-coumaric acid (A), ferulate monomers (B) and ferulate dimers (C). Ferulate monomers = trans- ferulic+ cis-ferulic acid. Ferulate dimers = 8-0-4’-diferulate + 5–5’ diferulate + 8-5cyclic diferulate (benzo form) + 8–5’-diferulate + an unknown ferulate dimer. Mean ± SEM (n = 2). Different letters indicate significant differences from the control (Tukey’s = 0.05).

Constitutive FAE expression either in the Golgi or in the apoplast also resulted in significant reductions in the level of cell wall esterified ferulates and diferulates in roots compared with control. Ferulate monomers were significantly reduced up to 50% (P < 0.0001) and 40% (P <0.0001) in Golgi (T28 and T29) and apoplast (T27 and T27R) targeted FAE plants compared with controls (Fig 10B). As for ferulate dimers, FAEA expression resulted in a reduction of up to 40% (P = 0.0019) and (P = 0.0023) in plants T27 and T28 respectively compared with control (Fig 10C). The level of *p-*coumaric acid in the roots of three of the four FAE expressing plants, was not significantly affected but was reduced in plant T27R (Fig 10A).

These results strongly suggest that the reduced ferulate deposition in FAE-expressing plants may be responsible for the changes in root growth and development described previously.

### Anatomical phenotypes

**Fig 11:**
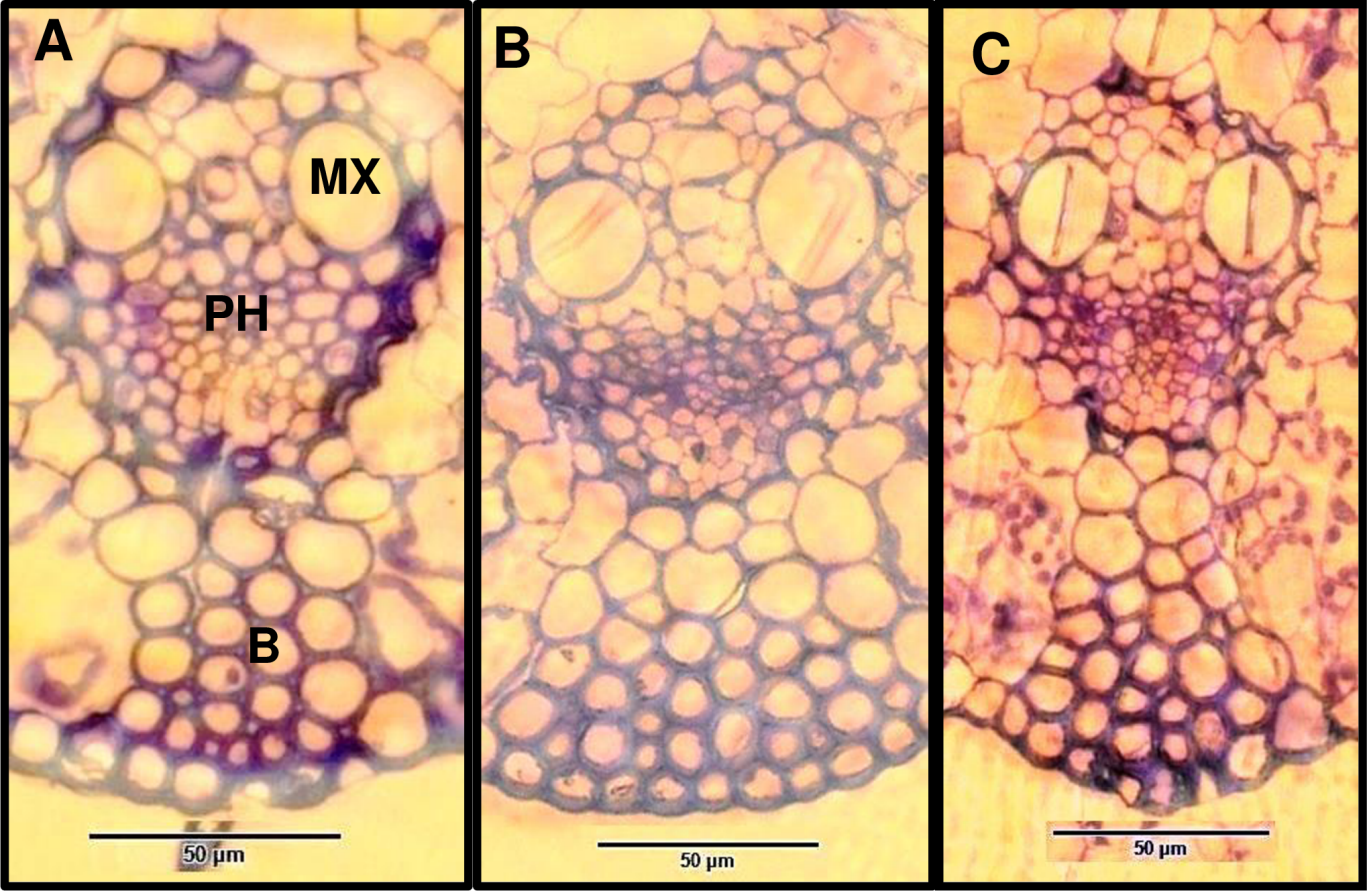
Transverse sections through the central vascular bundles of elongating leaf blades, 10 cm distal to the ligula, stained with toluidine blue. Control (B), apoplast (T27) (A) and Golgi (T28) (C). The metaxylem element (MX), abaxial fiber bundle (B) and phloem (PH), are indicated. Bar = 50 μm.

Microscopic examination of transverse sections through vascular bundles of the leaves of two of the transgenic lines (T27 and T28) when compared with control leaves (Fig 11A–11C) showed no evidence of vessel deformation. However, the sections indicate a reduced number of rows of abaxial fiber bundles in FAE expressing leaves.

### Senescence phenotypes and turnover of cell wall components in FAE expressing plants

The presence of FAE activity during leaf senescence (Fig 1) raises the question as to the extent to which cell wall arabinoxylans may become more easily remobilised during leaf senescence from cells with reduced arabinoxylan ferulate crosslinking. To address this question we first determined the fate of cell wall ferulates as the plants matured and then senesced.

#### Levels of cell wall ether+ester and ester linked HCAs in leaves of different ages

**Fig 12:**
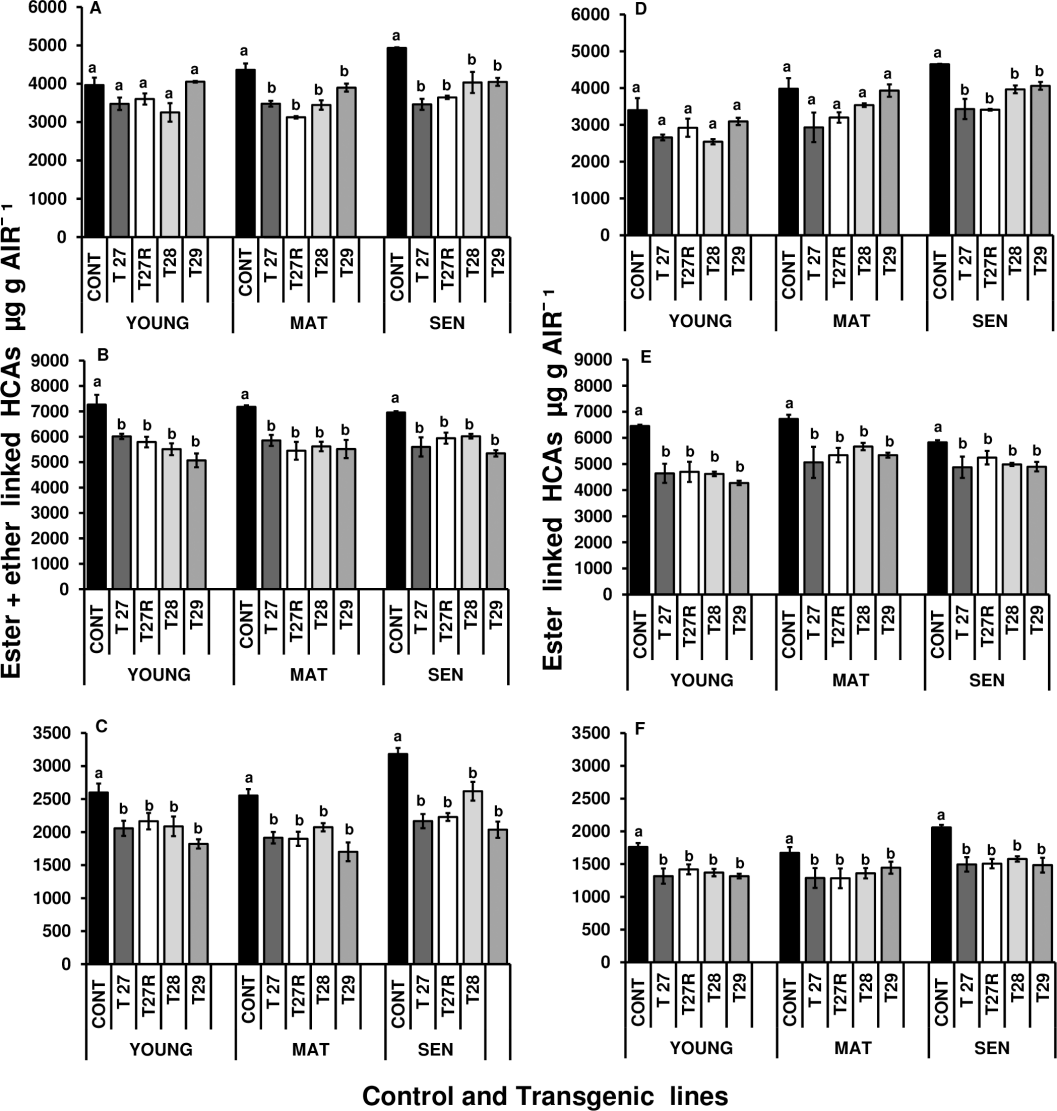
Ether+ ester (A, B, C) and ester linked (D, E, and F). *p*-coumaric acid (A, D), ferulate monomers (B, E) and ferulate dimers (C,F) in isolated cell wall AIR of young, mature and senescing leaves of control and apoplast (T27 and T27R) or Golgi (T28 and T29) FAE expressing plants. Ferulate monomers = trans- ferulic + cis-ferulic acid. Ferulate dimers = 8-0-4’-diferulate + 5–5’diferulate + 8-5cyclic diferulate (benzo form) + 8–5’-diferulate + an unknown ferulate dimer. Mean ± SEM (n = 3). Columns with different letters indicate statistically significant differences (P < 0.05).

Total ester+ether *p*CA levels were significantly lower in mature (P < 0.0001) and senescing (P < 0.021) leaves from apoplast and Golgi targeted FAE plants than in control leaves, but this was less pronounced for ester linked *p*CA (Fig 12A and 12D). Although ether-linked *p*CA has not been described, we found that harsher alkaline treatments used to break ether bonds released more *p*CA. The reduction in the level of ether+ester linked ferulate monomers was also significantly higher in FAE expressing lines at young, mature and senescence stages of development, (P = 0.0014; P = 0.0045; P < 0.0001 respectively) than in control leaves (Fig 12B). The effect was equally pronounced for ether+ester linked dimers at young, mature and senescing stages of development (P < 0.0001; P = 0.000; P < 0.0001 respectively) (Fig 12C). Similarly, ester-linked cell wall ferulate levels in young, mature and senescing leaves of FAE expressing lines showed a significant reduction in both monomers (P = 0.0025; P = 0.0006; P = 0.0016) and dimers (P = 0.0074; P = 0.0124; P < 0.0001 respectively) compared with control leaves (Fig 12E and 12F).

However, there was no evidence for enhanced turnover of either ester or ether linked monomeric or dimeric cell wall HCAs on leaf senescence in FAE expressing plants.

#### Levels of cell wall arabinoxylans in leaves of different ages

**Fig 13:**
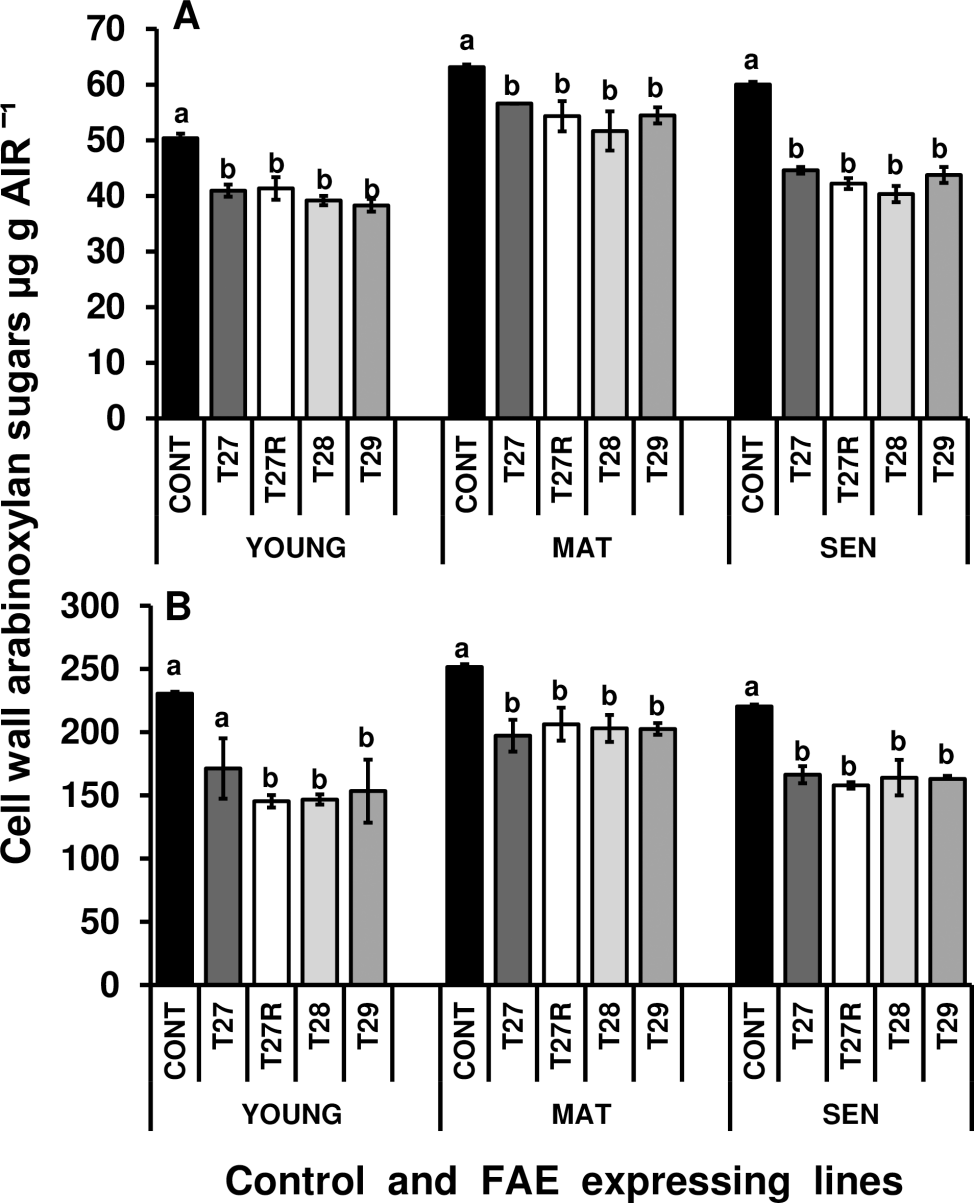
Arabinose (A) and xylose (B) composition of isolated cell wall AIR extracted from young, mature and senescing leaves of control and FAE expressing plants. Mean ± SEM (n = 3). Columns with different letters indicate statistically significant differences (P < 0.05).

Analysis of the arabinoxylan content of extracted cell walls from control and FAE expressing plants showed that mature control leaves lost 12.0% xylose and 8.0% arabinose upon senescence, whereas FAE expressing lines lost between 14.0 to 23.0% xylose and 11.0 to 19.0% arabinose (Fig 13) suggesting a senescence induced increased turnover of AX in FAE expressing leaves with reduced levels of cell wall HCAs.

However, mature leaves of FAE expressing lines contained between 17.9 and 22% less xylose and between 10.0 and 19.0% less arabinose than control mature leaves while senescing FAE expressing lines contained between 25.0 to 28.0% less xylose and between 26 to 33% less arabinose than control leaves. Pairwise comparisons between FAE expressing lines and controls showed significantly reduced levels of arabinose in young, mature and senescing leaves of plants T27 (P < 0.001; P = .0406; P = 0.001), T27R (P < 0.001; P = 0.0074; P < 0.001), T28 (P < 0.001; P = 0.0015; P < 0.0001) and T29 (P < 0.001; P = 0.0186; P < 0.0001)(Fig 13A). Xylose, levels in mature and senescing leaves were also statistically lower in all FAE expressing lines T27 (P = 0.0041 and P < 0.0001), T 27R (P = 0.0287 and P < 0.0001), T 28 (P = 0.0182 and P < 0.0001), T29 (P = 0.0417 and P < 0.0001) compared with control levels (Fig 13B), which provides some initial evidence for higher rates of turnover of cell wall arabinoxylans on leaf senescence in FAE expressing plants.

## Discussion

Previous studies have demonstrated the effectiveness of reducing the level of cell wall feruloylation in grasses by expression of a ferulic acid esterase from *Aspergillus niger* to different cell compartments [33, 34, 30]. As the specific roles of cell wall feruloylation in various plant processes has been established largely by indirect experiments, we have used these FAE expressing plants to test the hypothesized roles of cell wall feruloylation in plant growth and cell wall turnover.

The present results suggest that the observed reductions in leaf length and width in FAE expressing leaves may be a consequence of higher relative segmental cell elongation rates in FAE expressing leaves compared with controls indicating that FAE expressing cells were expanding faster than controls.

The increased growth rate could simply be due to lower rates of ferulate incorporation allowing cells to expand faster than controls, but then when they reach a critical ferulate level, they stop expanding by the same mechanism described by McAdam and Grabber (2002) [23] where leaf elongation decelerated as 8–5-, 8–O–4-, 8–8-, and 5–5-coupled diferulic acids accumulated in cell walls. The quantification of total cell wall (ether- and ester-linked) ferulate monomers and dimers along the leaf showed that accretion of ferulates, diferulates and *p*-coumaric acid, which is primarily associated with lignification in grass cell walls, reached the highest levels in FAE expressing leaves 20–30 mm from the ligula but in control leaves between 50–90 mm from the ligula. The earlier incorporation of *p*-coumarate into the cell walls may indicate that lignification is also occurring at an earlier stage of development but at a lower level in FAE expressing leaves than in control leaves. Interestingly, ferulate levels were quite similar between FAE expressing and control leaves from 10 to 20 mm from the ligula but significantly higher in control leaves from 20 mm from the ligula onwards. Graminaceous leaves are linear gradients of cell development with leaf age, with the youngest cells located at the base of the leaf [48, 49]. As cell wall ferulates were measured in leaves of FAE expressing and control leaves of similar lengths, this is likely to reflect a more advanced stage in FAE expressing leaves than in control leaves. In fact control leaves remained in a young-rolled stage until they reached three-quarters of their total length while FAE expressing leaves opened either while emerging or shortly after.

The finding that the chlorophyll levels measured in FAE expressing leaves of plant T27R decreased faster than in control leaves also supports the observation that leaves in FAE expressing plants, with reduced levels of cell wall ferulates, extended and matured before the controls. This is also supported by the finding that growth within the elongation zone of leaf blades with reduced levels of cell wall ferulates was closer to the ligula, with the maximum RSE rate occurring 9 mm above the ligula for FAE expressing leaves but at 12 mm from the ligula in control leaves. In addition, the elongation zone in FAE expressing leaves was longer (36 mm), than in control plants (30 mm) taken from the point where control leaves stopped extending.

Previous studies have associated cessation of cell expansion with formation of cell wall ferulate cross-linking and with increases in cell wall peroxidase activity in both tall fescue [23] and developing rice shoot [50]. It has been suggested [51] that by catalysing ferulate cross-linking, peroxidase rigidifies the cell wall and restricts growth. In the present study it was observed that the total POX activity in FAE expressing leaves increased to a higher level with increasing distance from the ligula. It is possible that conversion of soluble phenolics, released by FAE, into hydrophobic quinones catalysed by POX, is also contributing to early growth cessation in FAE expressing leaves. It is also possible that the higher peroxidase level in FAE expressing plants is catalysing earlier incorporation or earlier dimerization in FAE leaves which prevents expansion earlier but at a lower ferulate level than in controls.

In addition to a reduced leaf length and width, FAE expression also affected the size of leaf epidermal cells, which were significantly smaller in FAE expressing leaves compared with control leaves indicating that the expansion of epidermal cells was curtailed earlier than in control leaves. This may be due to the observations that both ether and ester linked monomeric HCAs and ferulate dimerization stopped earlier in FAE expressing leaves but at a lower level than controls. It has been shown [52] that slender barley mutants had increased leaf extension rates, longer leaves and longer epidermal cells. While the mechanisms responsible for altered leaf length in FAE expressing plants and in slender barley mutants are likely to be different, both observations suggest that changes in leaf length may be attributed, at least in part, to changes in individual cell length. As cell wall deposition and metabolism affects cell size and shape, and dwarfism quite often correlates with changes in cell morphogenesis and propagation, as has been shown in *Arabidopsis* [53], it is possible to speculate that cell wall ferulates and diferulates may affect plant growth and development by having a significant impact on either the progression of cell division or on the proportion of cells involved in cell division, via its role in wall assembly by cross-linking cell wall arabinoxylans, and that ferulate levels may alter the levels of AX being deposited in the cell wall.

It is possible that feruloylation is important or crucial for normal AX cell wall deposition so that disturbing the level of cell wall feruloylation, results in altered cell wall biogenesis and/or turnover. To support this theory we have also shown that FAE expression and consequent reduction in the level of ester and ether linked cell wall ferulates resulted in a significant increase in the loss of xylose and arabinose on senescence compared with senescing control leaves. As found previously [15–16], and in this study, the level of arabinosyl substitution decreases considerably when cells stop elongating with a corresponding increase in ferulated arabinosyl units. However, in FAE expressing plants, the level of ferulates and AX were significantly lower, and the ratio of xylose to arabinose was higher compared with controls indicating a turnover of arabinose-containing polymers. This suggests that FAE expression by removing ferulates from arabinose may make arabinose more likely to be recycled from the wall. Reduced levels of ferulate-dimers in FAE expressing leaves may also allow for lesser re-enforcement in the form of cross-links causing the arabinoxylan to be exposed to degradative enzymes and/or formation of shorter chains. This would not only reduce the number of sugar residues that can be added to a growing chain, but also would in effect reduce the length to which the leaf or root can grow.

The impact of a reduced level of cell wall feruloylation was also demonstrated by a significant reduction in root development and stresses the impact ferulate levels are likely to have on plant adaptation to drought conditions as the ability of a plant to survive under such conditions will at least in part depend on its ability to capture water through root exploration deep into the soil [54, 55].

It is known that mutants with abnormal xylan generally have a collapsed xylem phenotype since impaired vessel walls cannot withstand the high negative pressure generated by transpirational pull. This defect leads to a problem with water transport to photosynthetically active tissues and subsequently growth reduction and decrease in biomass accumulation [56–59] however, xylem morphology in FAE expressing leaves showed no evidence of xylem collapse.

In summary, the present study provides further evidence for cell wall ferulates as important players in plant growth as reduction of cell wall feruloylation negatively affected leaf and root growth, cell size and AX turnover on senescence. By cross-linking cell wall arabinoxylans, it is possible that ferulates can alter the level of AX being deposited in the cell wall and as such they are likely to be important or crucial for normal AX cell wall deposition. Taken together, the present study gives more support for the role of ferulates in two plant processes: plant growth and cell wall turnover.

## Conclusion

We have analysed the effect of manipulating the level of cell wall feruloylation in two plant processes, that of leaf growth and cell wall structure in tall fescue using transgenic plants expressing a ferulic acid esterase. FAE expression led to shorter and narrower leaves, higher relative segmental elongation rates, earlier overall growth rate, and shorter epidermal cells. Observations that both ether and ester linked monomeric HCAs and ferulate dimerization stopped earlier in FAE expressing leaves but at a lower level than in controls, and HCA deposition started to slow down when peroxidase levels increased, support the hypothesis that the timing of HCA deposition in the expanding cell walls controlled partially by the onset of peroxidase activity in this region is an important mechanism controlling leaf morphology. Also if we take the level of ester+ether linked *p*CA as a proxy for lignification, it appears that lignification also occurs at an earlier stage of development but at a lower level in FAE expressing leaves than in control leaves. It would appear therefore that one of the possible mechanism for controlling overall leaf morphology such as leaf length and width in grasses, where leaf morphology is highly variable between species, may be the timing of HCA deposition in the expanding cell walls as they emerge from cell division into the elongation zone, controlled partially by the onset of peroxidase activity in this region.

## Supporting information

S1 Fig Soluble peroxidase activity (A) and ionically bound peroxidase activity (B) in 0.5 cm sections along 14–16 cm leaves staring from the elongation zone of control and apoplast (T27 + T27R) or Golgi (T28+ T29) FAE expressing plants. Leaf sections (120–130) were pooled by location along the leaf blade from 8–9 tillers of 2 to 3 replicates per line from 2 independent FAE expressing plants.
(TIF)

S1 Table Average epidermal cell area (μm^2^) of the abaxial epidermis of fully expanded leaves of control and apoplast (T27 and T27R) or Golgi (T28 and T29) FAE expressing plants. Mean ± SEM n = 10 replicates of 10 leaves per plant, 1 cm from leaf tip in rows adjacent to the leaf midrib.
(DOCX)

S2 Table Average epidermal cell length (μm) of the abaxial epidermis of fully expanded leaves of control and apoplast (T27 and T27R) or Golgi (T28 and T29) FAE expressing plants. Mean ± SEM n = 10 replicates of 10 leaves per plant, 1 cm from leaf tip in rows adjacent to the leaf midrib.
(DOCX)

S3 Table Average epidermal cell width (μm) of the abaxial epidermis of fully expanded leaves of control and apoplast (T27 and T27R) or Golgi (T28 and T29) FAE expressing plants. Mean ± SEM n = 10 replicates of 10 leaves per plant, 1 cm from leaf tip in rows adjacent to the leaf midrib.
(DOCX)

S4 Table Average epidermal cell number per unit area at the base, middle and tip, of the abaxial epidermis of fully expanded leaves of control and apoplast (T27 and T27R) or Golgi (T28 and T29) FAE expressing plants. Mean ± SEM n = 10 replicates of 10 leaves per plant, 1 cm from leaf tip in rows adjacent to the leaf midrib.
(DOCX)

## Abbreviations

AIR: Alcohol Insoluble Residue
AX: Arabinoxylan
FA: Ferulic acid
FAEA: Ferulic acid esterase A
GAX: Glucuronoarabinoxylan
HCAs: Hydroxycinnamic acids
HPAEC: Anion exchange chromatography
HPLC: High performance liquid chromatography
*p*CA: *p*-coumarate
POX: Peroxidase
TFA: Trifluoroacetic acid
