# Supplementary figures and images for: Reducing cell wall feruloylation by expression of a fungal ferulic acid esterase in Festuca arundinacea modifies plant growth, leaf morphology and the turnover of cell wall arabinoxylans
Source: PLoS One. 2017 Sep 21;12(9):e0185312. doi: 10.1371/journal.pone.0185312 (PMC5608373; doi:10.1371/journal.pone.0185312)

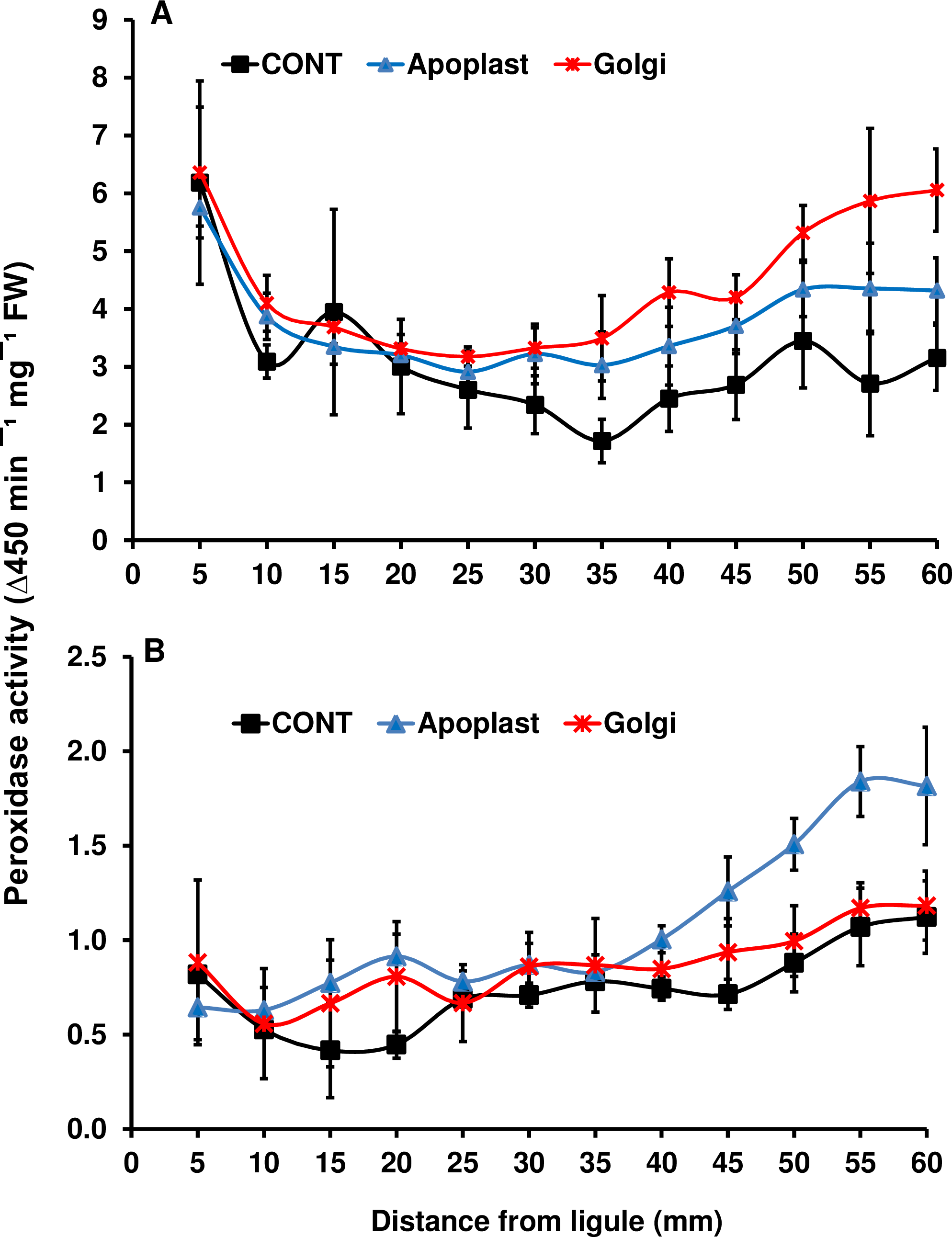

Supplement: S1 Fig — Soluble peroxidase activity (A) and ionically bound peroxidase activity (B) in 0.5 cm sections along 14–16 cm leaves staring from the elongation zone of control and apoplast (T27 + T27R) or Golgi (T28+ T29) FAE expressing plants. Leaf sections (120–130) were pooled by location along the leaf blade from 8–9 tillers of 2 to 3 replicates per line from 2 independent FAE expressing plants. (TIF) [file pone.0185312.s001.tif]
