# Supplementary material for: Reducing cell wall feruloylation by expression of a fungal ferulic acid esterase in Festuca arundinacea modifies plant growth, leaf morphology and the turnover of cell wall arabinoxylans
Source: PLoS One. 2017 Sep 21;12(9):e0185312. doi: 10.1371/journal.pone.0185312 (PMC5608373; doi:10.1371/journal.pone.0185312)
Supplement: Supplementary file 2 [file pone.0185312.s002.docx]

			Area (µm²)		
**REPLICATES**	**CONT**	**T 27**	**T 27R**	**T28**	**T29**
1	3593.88±268	2924.13±28	2012.69±27	2459.75±22	2549.03±11
2	3762.06±34	2492.14±71	2430.88±28	2334.04±16	3057.25±25
3	4439.71±36	2604.99±17	2322.85±14	2446.66±22	2924.58±26
4	4546.05±63	2554.44±12	2377.47±13	2264.74±19	2391.52±14
5	3522.21±36	2685.44±32	2524.40±20	2539.00±17	2361.77±18
6	4513.48±17	2293.26±18	2938.42±22	2197.69±19	2868.36±19
7	3761.58±59	2288.74±26	2199.01±12	2549.12±19	2579.95±20
8	4077.03±20	2326.20±20	2597.51±27	2583.38±59	2825.07±31
9	4367.26±23	2275.97±16	2651.54±17	2302.75±17	2713.46±37
10	3572.60±12	2489.74±53	3045.61±14	3050.91±43	2400.15±70
**TOTAL MEANS**	**4015.58±132**	**2493.47±66**	**2510.03±100**	**2472.80±76**	**2667.11±78**
