# Supplementary material for: Reducing cell wall feruloylation by expression of a fungal ferulic acid esterase in Festuca arundinacea modifies plant growth, leaf morphology and the turnover of cell wall arabinoxylans
Source: PLoS One. 2017 Sep 21;12(9):e0185312. doi: 10.1371/journal.pone.0185312 (PMC5608373; doi:10.1371/journal.pone.0185312)
Supplement: Supplementary file 3 [file pone.0185312.s003.docx]

	Length (µm)				
**REPLICATES**	**CONT**	**T 27**	**T 27R**	**T28**	**T29**
1	290.08±16	196.09±19	168.76±14	194.04±15	186.26±80
2	289.80 ±22	142.73±8	180.87±15	170.98±70	222.34±22
3	307.36±31	197.70±22	181.42±13	201.07±16	198.01±21
4	281.55 ±25	150.78±9	156.81±13	189.01±15	186.98±15
5	263.99±12	208.32±19	167.04±16	213.55±14	184.06±11
6	270.90±60	206.50±15	225.29±26	191.82±19	221.09±14
7	274.49 ±13	182.30±23	184.54±15	199.33±12	176.79±11
8	265.84±10	166.74±12	201.40±13	219.22±18	208.95±17
9	307.31±16	163.23±10	172.59±10	186.98±17	231.57±30
10	245.94±80	216.27±31	200.84±90	174.59±13	175.20±40
**TOTAL MEANS**	**280.49±70**	**173.07±30**	**184.30±50**	**193.99±50**	**195.83±13**
