# Supplementary material for: Reducing cell wall feruloylation by expression of a fungal ferulic acid esterase in Festuca arundinacea modifies plant growth, leaf morphology and the turnover of cell wall arabinoxylans
Source: PLoS One. 2017 Sep 21;12(9):e0185312. doi: 10.1371/journal.pone.0185312 (PMC5608373; doi:10.1371/journal.pone.0185312)
Supplement: Supplementary file 4 [file pone.0185312.s004.docx]

	Width (µm)				
**REPLICATES**	**CONT**	**T 27**	**T 27R**	**T28**	**T29**
1	12.63±0.8	15.09±0.7	13.81±1.5	12.51±0.5	12.32±0.7
2	13.97±0.4	16.83±0.6	12.38±1.4	12.54±0.6	13.14±0.4
3	14.79±0.5	13.32±0.4	12.01±1.4	12.18±0.8	14.14±0.4
4	16.33±0.9	15.65±0.4	15.77±1.0	12.01±0.3	11.83±0.5
5	12.88±0.4	11.58±0.5	14.59±1.2	12.16±0.4	11.79±0.5
6	15.81±0.5	10.61±0.2	14.59±0.5	13.04±0.3	12.14±0.3
7	13.69±0.7	12.77±0.4	12.19±0.6	12.90±0.8	13.75±0.5
8	14.68±0.3	13.53±0.8	12.75±0.9	12.97±1.6	16.13±0.6
9	13.98±1.0	14.26±0.5	15.28±0.6	11.87±0.5	14.63±0.5
10	12.12±1.0	11.40±1.1	15.07±0.4	16.90±1.0	14.74±0.7
**TOTAL MEANS**	**14.09±0.4**	**13.5±0.6**	**13.84±0.4**	**12.96±0.5**	**13.46±0.4**
