# Supplementary material for: Reducing cell wall feruloylation by expression of a fungal ferulic acid esterase in Festuca arundinacea modifies plant growth, leaf morphology and the turnover of cell wall arabinoxylans
Source: PLoS One. 2017 Sep 21;12(9):e0185312. doi: 10.1371/journal.pone.0185312 (PMC5608373; doi:10.1371/journal.pone.0185312)
Supplement: Supplementary file 5 [file pone.0185312.s005.docx]

	Number 10^6 µm^{-2}			Number 10^6 µm^{-2}		
	CONT			T27R		
**REPLICATES**	**BASE**	**MID**	**TIP**	**BASE**	**MID**	**TIP**
1	1911.11±111	1933.33±66	1666.66±176	1911.11±160	1622.22±88	1666.66±101
2	2088.88±256	1800.00±101	1777.77±173	2022.22±58	1622.22±22	1577.77±22
3	1911.11±160	1977.77±58	1844.44±155	2111.11±117	1755.55±173	1555.55±88
4	1977.77±96	1955.55±88	1822.22±155	2066.66±101	1577.77±117	1577.77±22
5	1955.55±58	2066.66±38	1933.33±101	2022.22±22	1422.22±44	1555.55±22
**TOTAL MEANS**	**1968.88±60**	**1946.66±36**	**1808.88±63**	**2026.66±43**	**1600.00±48**	**1586.66±26**
